# Cytokine responses to SARS-COV2 infection in mother-infant dyads: a systematic review and meta-analysis

**DOI:** 10.3389/fped.2023.1277697

**Published:** 2023-10-17

**Authors:** Samhita Jain, Isabel Elaine Allen, Dongli Song, Xianhua Piao

**Affiliations:** ^1^ Division of Neonatology, Department of Pediatrics, University of California, San Francisco, San Francisco, CA, United States; ^2^ Department of Epidemiology and Biostatistics, University of California, San Francisco, San Francisco, CA, United States; ^3^ Department of Pediatrics, Division of Neonatology, Santa Clara Valley Medical Center, San Jose, CA, United States; ^4^ Department of Pediatrics, Stanford University School of Medicine, Stanford, CA, United States; ^5^ Newborn Brain Research Institute, University of California, San Francisco, San Francisco, CA, United States; ^6^ Weill Institute for Neuroscience, University of California, San Francisco, San Francisco CA, United States

**Keywords:** SARS-CoV2, pregnancy, cytokines, mother-infant dyad, cord blood

## Abstract

**Background:**

The COVID-19 pandemic has affected a significant number of pregnant women worldwide, but studies on immune responses have presented conflicting results. This study aims to systematically review cytokine profiles in pregnant women with SARS-CoV-2 infection and their infants to evaluate immune responses and potential transplacental transfer of cytokines.

**Materials and methods:**

A comprehensive search of 4 databases was conducted to identify relevant studies. Inclusion criteria included studies measuring individual cytokines in pregnant women and/or their neonates. Studies were evaluated for quality, and data were extracted for analysis. Meta-analyses were performed using the random-effects model.

**Results:**

Seventeen studies met the inclusion criteria, including data from 748 pregnant women and 287 infants. More than three of these studies evaluated data of 20 cytokines in maternal serum, and data of 10 cytokines was available from cord blood samples. Only the serum level of CXCL10 was significantly up-regulated in SARS-CoV-2 positive pregnant women (*n* = 339) compared to SARS-CoV-2 negative pregnant women (*n* = 409). Subset analysis of maternal samples (*n* = 183) collected during the acute phase of COVID-19 infection showed elevated CXCL10 and IFN-γ. No significant differences in cytokine levels were found between cord blood samples collected from infants born to mothers with (*n* = 97) and without (*n* = 190) COVID-19 during gestation. Subset analysis of cord blood samples collected during the acute phase of maternal infection was limited by insufficient data. The heterogeneity among the studies was substantial.

**Conclusion:**

The findings suggest that maternal cytokines responses to SARS-CoV-2 infection during pregnancy are not significantly dysregulated, except for CXCL10 and IFN-γ during the acute phase of illness. No evidence of increased cytokine levels in cord blood samples was observed, although this could be impacted by the time period between initial maternal infection and cord blood collection. These results provide some reassurance to parents and healthcare providers but should be interpreted cautiously due to study variations and limitations.

## Introduction

Pregnancy involves changes in the immune system of women to ensure tolerance of the fetus, which may make pregnant women more vulnerable to any infection. COVID-19 has a significant negative impact on pregnancy. Compared with non-pregnant women of a similar age, pregnant women are at a higher risk of developing severe COVID-19, requiring intensive care unit admission, mechanical ventilation or extracorporeal membrane oxygenation support, and even death (1–4). Additionally, there is an increased risk of obstetric complications and adverse birth outcomes, such as maternal hypertension and preterm birth in mothers with COVID-19 during pregnancy s (3–7).

Current data suggests that vertical transmission of the SARS-CoV-2 virus to the fetus is relatively rare (7). However, studies have provided evidence of the transplacental passage of immune mediators, including virus-specific antibodies and cytokines, in response to maternal infections, including SARS-CoV-2 (7–9). Previous research on influenza virus pandemics has demonstrated a 2–3 fold increased risk of autism spectrum disorder and schizophrenia in offspring of women who had influenza during pregnancy (10–12). Moreover, the injection of Poly I:C, a synthetic double-stranded RNA viral mimetic, in rodent models has been demonstrated to induce substantial maternal immune activation and lead to behavioral phenotypes resembling autism spectrum disorder (ASD) and Schizophrenia (13–15). This raises concerns about the long-term neurodevelopmental outcomes in children born to mothers with SARS-CoV-2 infection during pregnancy. In a global context where around 140 million live births take place each year, and with a prevalence of up to 15% of SARS-CoV-2 positivity among pregnant women in urban areas, it is estimated that as many as 20 million children could be exposed to maternal COVID-19 infection during pregnancy annually (16, 17). This situation raises significant concerns, especially in regions where pregnant populations have low COVID-19 vaccination rate. There are limited reports on the long-term neurodevelopment of children who were born to mothers with SARS-CoV-2 infection during pregnancy. An electronic health record (EHR)–based 12-month follow-up study of over 7,000 deliveries, including more than 200 COVID-19-exposed pregnancies, suggested that prenatal SARS-CoV-2 infection is associated with an increased risk of neurodevelopmental disorders in offspring (18). A prospective cohort study using a standardized observer-based assessment showed normal neurodevelopment at 5–11 months in infants born to mothers with asymptomatic or mild SARS-CoV-2 infection during pregnancy (19). Two recent meta-analyses found that gestational exposure to SARS-CoV-2 did not change overall neurodevelopment in the first year of life (20, 21) except for negative effects on fine motor and problem-solving skills (21). Large scale and longer neurodevelopment follow-up studies are needed.

There exist various plausible mechanisms through which maternal infection with SARS-CoV-2 could impact the developing fetal brain. In addition to immune factors, non-immune mediated effects could potentially impact fetal brain development, such as direct viral infection of fetal neurological cells through transmission across the placenta and compromised placental function, which can lead to adverse pregnancy outcomes and an elevated risk of neurological harm, such as fetal growth restriction, premature birth, or placental abruption. Recent investigations, involving both human subjects and animals, into the consequences of maternal immune activation resulting from viral and bacterial infections during pregnancy have underscored the role of maternal cytokines in potentially contributing to the pathogenesis of preterm birth and negative neurodevelopmental outcomes in the offspring (15, 22–24). While a few studies have examined changes in peripheral blood cytokine levels in response to acute SARS-CoV-2 infection during pregnancy (25–41), their findings are not entirely consistent, possibly due to limited sample sizes, variations in study design, or other factors that can influence cytokine levels, such as timing of sample collection, treatment of infection and inflammation. Therefore, the aim of this study is to systematically review the cytokine profiles of mothers with SARS-CoV-2 infection during pregnancy. Additionally, we will review the cord blood cytokine profiles of infants exposed to SARS-CoV-2 during gestation to evaluate the potential transplacental transfer of these cytokines.

## Materials and methods

Our study adheres to the Preferred Reporting Items for Systematic Reviews and Meta-Analyses (PRISMA) guidelines for systematic reviews (42). In collaboration with a medical librarian, we conducted a comprehensive search of the PUBMED, EMBASE, Web of Science, and Coronavirus Research Databases to identify relevant literature published until August 2022. The search strategy is described in detail in Supplementary File S1. Two reviewers (SJ and EIA) independently screened the titles and abstracts of the articles identified through the search. Any disagreements regarding the inclusion or exclusion of a study were resolved through consensus, and if necessary, consultation with a third author (XP).

The following inclusion criteria were applied to select articles for this systematic review and meta-analysis: all studies that evaluated the measurement of individual cytokines in pregnant women and/or their neonates were included. For the control group, data on individual cytokine measurements from healthy pregnant women and their neonates were collected from the same selected studies. We only included studies evaluating maternal serum samples or cord blood samples. We considered studies of any design and from any period since the outbreak of SARS-COV2 commenced as eligible for inclusion. We established exclusion criteria, which entailed the removal of case reports pertaining to individual patient, literature reviews, studies involving nonhuman subjects, editorials, comments, and expert opinions from our analyses. The full texts of potentially relevant studies were carefully reviewed to determine their eligibility based on the inclusion and exclusion criteria. The final list of included studies was identified through consensus among the reviewers.

The reviewers then independently extracted data from the included studies. The extracted details included baseline information on the study population, the number of patients in each study group, the measured immunological indicators, and the methods used for testing. These details are presented in Table 1. We focused our subsequent analysis solely on immune mediators that had been investigated in at least three included studies. Mean values (in pg/ml) of individual cytokines were extracted from the manuscript texts, tables, supplementary data, source data, and figures of each study.

**Table 1:** Characteristics of included studies.

Source	Country	Study design/patient group	Sample size		Sample assessed	Cytokines assessed	Detection method
			Study cohort	Controls			
Boelig et al. (25)	USA	Retrospective cohort study, at at Thomas Jefferson University Hospital from March 2020 to July 2021	RT-PCR confirmed COVID19 pregnant women (anytime during pregnancy), *N* = 58	Pregnant women with no signs of COVID19, *N* = 142.	Maternal serum at delivery, Cord blood and placenta	1β, IL-8, IL-2, IL-4, IL-6, IL-10, IL-12p70, IL-13, IFNg, and TNFα	Meso Scale Diagnostics platform using the 10-plex human Proinflammatory panel kit
Brancaccio et al. (26)	Italy	Prospective observational case-control study, enrolled between January 2021 and June 2021	RT-PCR confirmed COVID19 pregnant women, *N* = 22	Pregnant women with no signs of COVID19, *N* = 22	Maternal samples collected at admission to COVID maternity ward	2 cytokines- IL-6, IL-8	ELISA on maternal serum and cord blood samples for IL6, 8.
Cerbulo-Vazquez et al. (27)	Mexico	Prospective observational case-control study	RT-PCR confirmed COVID19 pregnant women, *N* = 14	Pregnant women with no signs of COVID19, *N* = 13	Maternal serum samples collected at enrollment	IL-2, IL-4, IL-6, IL-10, TNF-α, IFN-*γ*, and IL-17a) and chemokines (CXCL8/IL-8, CXCL10/IP-10, CCL11/Eotaxin, CCL17/TARC, CCL2/MCP-1, CCL5/RANTES, CCL3/MIP-1a, CXCL9/MIG, CXCL5/ENA-78, CCL20/MIP-3a, CXCL1/GROa, CXCL11/I-TAC and CCL4/MIP-1b	Bead-based immunoassays (CBA kit, Cat. 560484, BD Pharmingen, San Diego, CA, USA; and LEGENDplex, Cat. 740003, BioLegend, San Diego, CA, USA, respectively
Chen et al. (28)	China	Retrospective single-center study, enrolled between Jan-April 2020	RT-PCR or specific Ab positive pregnant women, *N* = 11	Healthy pregnant women, *N* = 10	Maternal serum samples accessed from the specimen bank at Tongji Hospital, no timing specified	48 cytokines- FGFb, Eotaxin, GCSF, GM-CSF,IFNg, IL1B, IL1ra, IL1a, IL2ra, IL3, IL12(p40), IL16, IL2, IL4, IL5, IL6, IL7, IL8, IL9, GRO-a, HGF, IFN-a2, IL10, IL12(p70), IL13, IL15, IL17a, IP10, MCP1, MIG, b-NGF, SCF, SCGFB, SDF1A, MIP1A, MIP1B, PDGF-BB, RANTES, TNFa, VEGF, CTACK, MIF, TRAIL, IL18, M-CSF, TNFB	Measured using the 152 bio-plex pro human cytokine screening panel (Bio-Rad)
Chen et al. (29)	China	Retrospective single-center study, enrolled between Jan-May 2020	RT-PCR or specific Ab positive pregnant women, *N* = 16, 4 for IFN	Healthy pregnant women, *N* = 4	Maternal serum samples accessed from the specimen bank at Tongji Hospital, between 56 and 119 days after the symptom onset	IL-6, IL-1b, IL-2R, IL-8, IL-10, and TNF-α	ECLIA (electrochemiluminescence immunoassay) Roche Diagnostics, DiaSorin
DeBiasi et al. (30)	Italy	Case- control, cross-sectional, single-center study	RT-PCR confirmed COVID19 pregnant women, *N* = 14	RT-PCR negative healthy, age matched pregnant women, *N* = 28	Maternal serum samples obtained at enrollment	62 cytokines- G-CSF, PDGF-AA, EGF, PDGF-AB/BB, VEGF, GM-CSF, FGF, GRZB, IL- 1A, IL-1RA, IL-2, IL-27, IL-4, IL-6, IL-10, IL-13, TNF, IL-17C, IL-11, IL-18, IL-23, IL-6RA, IL-19, IFN-B, IL-3, IL-5, IL-7, IL-12p70, IL-15, IL-33, TGF-B, IFN-G, IL- 1B, IL-17, IL-17E, CCL3, CCL11, CCL20, CXCCL1, CXCL2, CCL5, CCL2, CCL4, CCL19, CXCL1, CXCL10, PD-L1, FLT-3, TACI, FAS, LEPTIN R, APRIL, OPN, BAFF, LEPTIN, BMP4, CD40 LIGAND, FAS LIGAND, BMP7, BMP2, and TRAIL	Luminex platform (Human Cytokine Discovery, R&D System, Minneapolis, MN
Febryanna et al. (31)	Indonesia	Case- control, cross-sectional, single-center study	RT-PCR confirmed COVID19 pregnant women, *N* = 25	RT-PCR negative healthy, pregnant women, *N* = 25	Maternal serum sample collected at admission for delivery	TNF-a	BD CBA (cytometric bead array)human Th1/Th2 Cytokinekit II CAT No. 551809
Garcia-Flores et al. (32)	USA	Case- control, cross-sectional, single-center study	RT-PCR confirmed COVID19 pregnant women, *N* = 12. 8 asymptomatic, 1 mild symptoms and 3 severe COVID-19 (requiring O2)	RT-PCR negative healthy, pregnant women, *N* = 11	Maternal blood samples were collected upon admission, mostly all term gestation and cord blood at delivery	20 cytokines- IFN-γ, IL-1β, IL-2, IL-4, IL-6, IL-8, IL-10, IL-12p70, IL-13, and TNFA (Pro-inflammatory Panel 1) or GM-CSF, IL-1α, IL-5, IL-7, IL-12/IL- 23p40, IL-15, IL-16, IL-17A, TNF-β, and VEGF-A	V-PLEX Pro-Inflammatory Panel 1 (human) and Cytokine Panel 1 (human) immunoassays (Meso Scale Discovery, Rockville, MD, USA)
Gee et al. (33)	UK	Case- control, cross-sectional, single-center study, May 2020-March 2021	RT-PCR confirmed COVID19 pregnant women, R/O (Infection within 2 weeks of delivery) *N* = 15, Recovered (nfection in early gestation) *n* = 14	RT-PCR negative throughout pregnancy, healthy, pregnant women, *n* = 15	Umbilical cord blood samples and paired maternal blood samples collected at the time of delivery	13 cytokines- IL-1β, IL-6, TNF, IP-10, CXCL8, IL-12p70, IFN-α2, IFN-*λ*1, IFN-λ2/3, GM-CSF, IFN-β, IL-10 and IFN-γ	13-plex LegendPlex human anti-virus response panel kit (BioLegend)
Rosen et al. (34)	USA	Observational study, single center, cross sectional study, March-April 2020	RT-PCR confirmed COVID19 in pregnant women at admission during 3rd trimester, *n* = 44	RT-PCR negative healthy, pregnant women in 3rd trimester, *n* = 25	Maternal blood samples drawn at admission for symptoms or delivery	7 cytokines- G-CSF, HGF, IL-18, IL-1Ra, IL-2Ra, IL-8, and IP-10	Quantikine ELISA kits from R&D Systems (Minneapolis, MN, USA)
Taglauer et al. (35)	USA	Prospective cohort study from July 2020 through June 2021	RT-PCR confirmed COVID19 anytime during pregnancy, *N* = 31	Contemporary RT-PCR negative healthy, pregnant women, *N* = 29	Maternal blood and cord blood/infant blood at the time of delivery	13 cytokines- IP-10, IL-1β, IL-6, TNF-α, IFN-λ1, IL-8, IL-12p70, IFN- α2, IFN-λ 2/3, GM-CSF, IFN- β, IL-10, and IFN-γ.	LEGENDplex assay (BioLegend)
Tanacan et al. (36)	Turkey	Prospective case-control study	Pregnant women with confirmed COVID-19 infection, *N* = 90	Gestational age-matched control group of healthy pregnant women, *N* = 90	Blood samples were collected from the participants along with the initial laboratory tests upon their first admission to the hospital.	5 cytokines- IFN γ, IL-2, IL-6, IL-10, and IL-17	ELISA kits by eBioscience, Thermo Fisher Scientific
Tartaglia et al. (37)	Italy	Prospective case-control study	COVID recovered Pregnant women (pCOV), *n* = 17	COVID recovered matched non- pregnant women (nCOV), *n* = 12	Maternal serum samples, no timing mentioned	11 cytokines, chemokines, and growth factors- GM-CSF, IFN-gamma, IL-1 beta/ IL-1F2, IL-2, IL-4, IL-5, IL-6, IL-10, IL-12 p70, IL-13, TNF-alpha, IL-17	Luminex Human Th1/Th2 11-plex Fixed Panel, Human IL-17 immunoassay, biotechne
Gonzalez-Mesa et al. (38)	Spain	Observational and prospective study, November 2020 to May 2021	RT-PCR confirmed COVID19 in pregnant women during labour or anytime during pregnancy, *n* = 79	None	Blood samples were collected from the mother at the time of labor and from the umbilical cord immediately after birth	3 cytokines- IL1b, IL6, and IFN-γ	ELISA
Briana et al. (39)	Greece	Prospective, observational study March 2020 and April 2021	Term infants born to previously healthy mothers; uncomplicated up to term pregnancies; positive for SARS-CoV-2 maternal nasopharyngeal swabs at delivery, negative neonatal RT-PCR, *N* = 40	None	Neonatal blood on Day 0	1 cytokine- IL6	ELISA
Liu et al. (40)	China	Prospective, observational study January 20 to March 3, 2020	Term newborns born to mothers with RT-PCR pos COVID-19, *N* = 51	None	Blood samples were collected within 3 days after birth for the detection of immunoglobulin levels, cytokine concentrations, and lymphocyte subsets.	6 cytokines- IFN-g, IL-2, IL-4, IL-6, IL-10, and TNF-a	FACS Calibur Flow Cytometer (BD Biosciences)
Zhong et al. (41)	China	Retrospective, observational study	RT-PCR confirmed COVID19 pregnant women, *N* = 36	RT-PCR negative pregnant women, *N* = 36 (No cytokines)	Blood sample collected at admission for delivery or illness	IL4, IL6, IL10, TNFa, IFNg	Flow Cytometry (CellGene Biotech Co)

### Statistical analysis

We collected the number of participants, the mean and standard deviation (SD) values for immune mediators from serum samples of pregnant women with and without COVID-19, as well as from cord blood samples of infants born to mothers with and without COVID-19 during pregnancy. We conducted a subset analysis of maternal serum samples collected within 2 weeks of COVID-19 diagnosis to capture acute and ongoing infection. COVID-19 infection was confirmed through a positive nasopharyngeal RT-PCR test result at any time during pregnancy in all studies. The control cohort comprised women with documented negative COVID-19 nasal swabs throughout their pregnancy. Women who received the COVID-19 vaccine were excluded from both the study and control cohorts. In cases where mean and SD data were not directly reported, we applied a previously established method to convert median and interquartile range (IQR) values to mean and SD. For articles presenting immunological signatures in figures, we extracted data by measuring the pixel positions of the electronic figures and calculating the actual values. For box plots, medians and ranges were used to derive means and SDs, while scatter plots provided individual values for the computation of means and SDs.

Random effects meta-analyses were performed for all immune mediators separately for mothers and infants and for subgroups. Forest plots were generated to illustrate the differences between the two groups. Due to potential variations in cytokine testing methods across studies, which can affect the pooling of means and differences for effect estimation, we calculated a dimensionless effect measure from each study for pooling purposes. The standardized mean difference (SMD) was computed using the means and SDs and used as the effect size. Heterogeneity was assessed using the I^2^ statistic. Publication bias was assessed using funnel plots and the Begg and Egger tests. All analyses were performed in Stata v.17.1 (StataCorp, College Station, TX).

## Results

**Figure 1:**
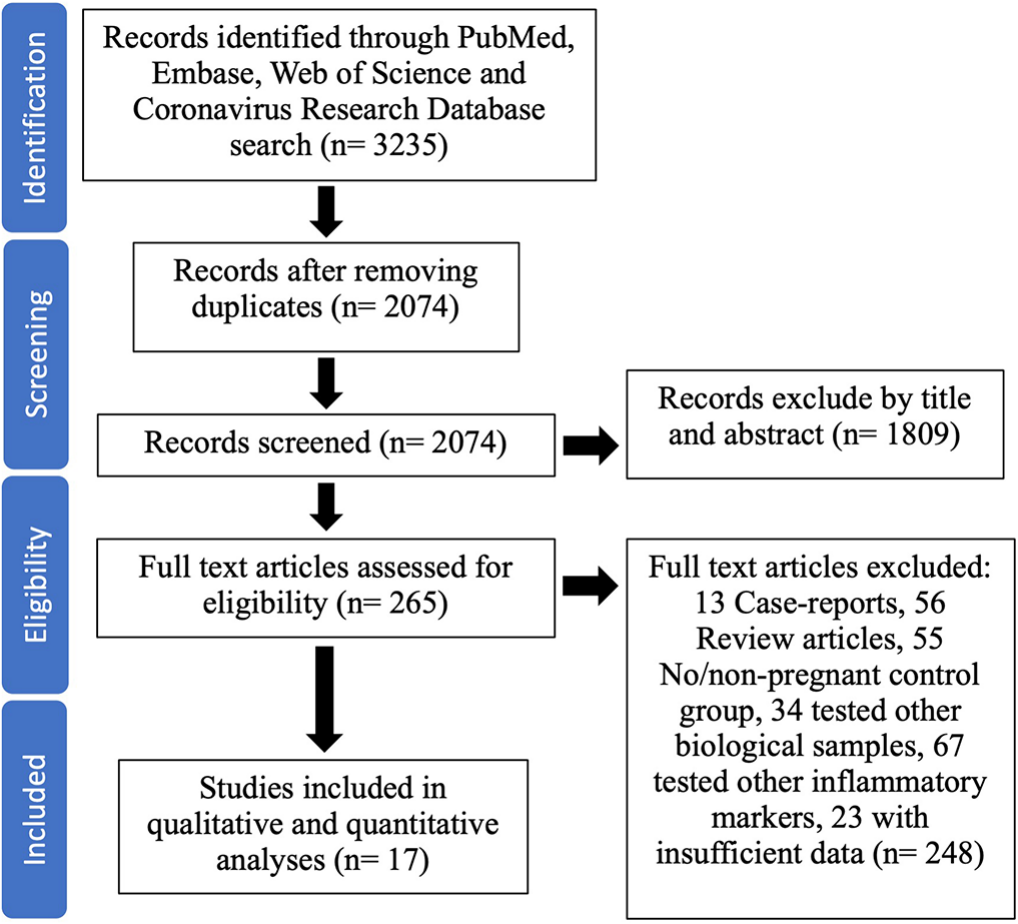
Flow diagram of the inclusion criteria for the study selection process.

A detailed flow diagram illustrating the study selection process and the number of studies selected is presented in Figure 1. Our literature search identified 3,235 reports up until 29 August 2022. After removing duplicates, we screened the titles and abstracts of the remaining 2,074 articles, further excluding 1,809 reports. Full texts of the remaining 263 potentially relevant studies were retrieved and evaluated for eligibility, leading to the exclusion of 246 studies from the meta-analysis for various reasons (detailed in Figure 1). Ultimately, 17 studies were included in the qualitative synthesis and collated for the meta-analysis (25–41). These studies were conducted in China (4), the United States (4), Italy (2), Greece (1), Indonesia (1), Mexico (1), Spain (1), and Turkey (1) (Table 1). All included studies reported individual cytokine levels in maternal and/or cord blood samples from pregnant individuals with and without COVID-19 infection and their infants, respectively.

The articles included data from 748 pregnant women, with 46% (*n* = 339) documented to have COVID-19 infection during pregnancy and 54% (*n* = 409) without documented COVID-19 infection. In all included reports, the study and control cohorts were matched for maternal age. The studies also included data from 287 infants, with 34% (*n* = 97) born to mothers with COVID-19 infection during pregnancy and 66% (*n* = 190) born to mothers without COVID-19 infection. More than three of these studies evaluated data on 20 cytokines in maternal serum, and data on 10 cytokines was available from cord blood samples. A comprehensive overview of the characteristics of the included studies for each immune mediator including baseline information, number of patients in each study group, measured immunological indicators and their outcome reporting measures, were extracted and summarized in Supplementary Table S1 for maternal samples and Supplementary Table S2 for cord blood samples.

In the subset analysis of maternal samples, only studies that reported cytokine levels during the acute phase of COVID-19 infection (i.e., sample collection and testing within 2 weeks of a documented positive nasal swab) were included. This subset analysis included data on 10 cytokines from maternal serum of 183 pregnant women with active COVID-19 infection and 214 pregnant women without COVID-19 infection. The study and control groups were age matched in all the included studies in the subset analysis as well. However, only 1 report (Tanacan et al) (36) had gestational age matched study and control cohorts from every trimester. It is important to note that 4 reports (26, 27, 32, 34) out of the remaining six (26, 27, 30, 32, 34, 35) in the subset analysis, primarily enrolled women with COVID 19 positive nasopharyngeal swab closer to delivery and thus the mean gestational age between the study and control groups were similar (∼38–39 weeks of gestation) in these studies. Similar details were extracted and summarized in Supplementary Table S3 for each study in the subset analysis. For these studies, only measurements acquired during the acute phase of infection were used for the analysis. Supplementary Table S3 provides an overview of the characteristics of the included studies for each immune mediator in the subset analysis.

**Figure 2:**
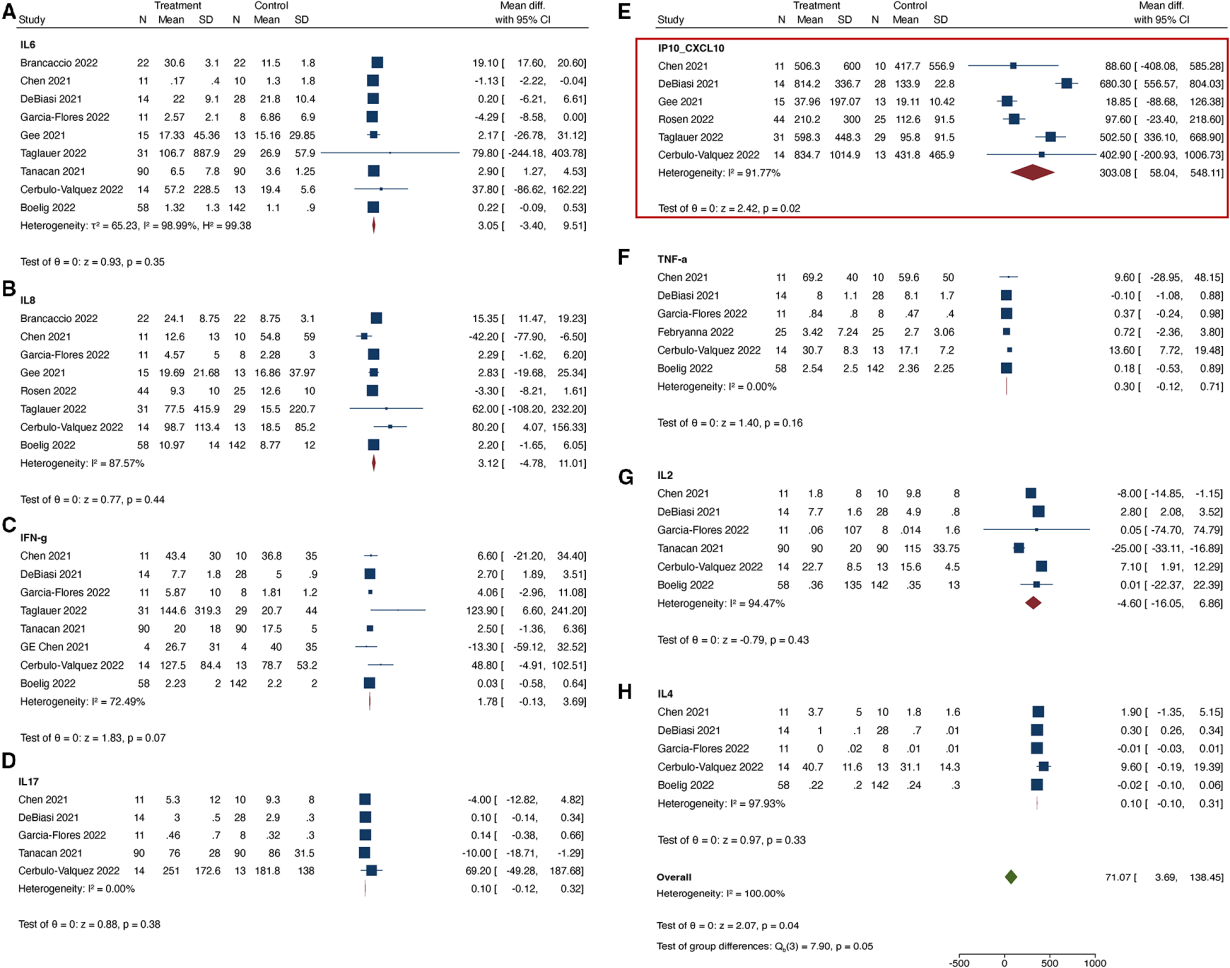
(**A–H**) Cytokines tested in maternal serum samples. In Figures 2–4, “Treatment” group refers to “COVID positive” and “Control” group refers to “COVID negative” pregnant individuals. All cytokines have been reported as mean values in pg/ml.

**Figure 3:**
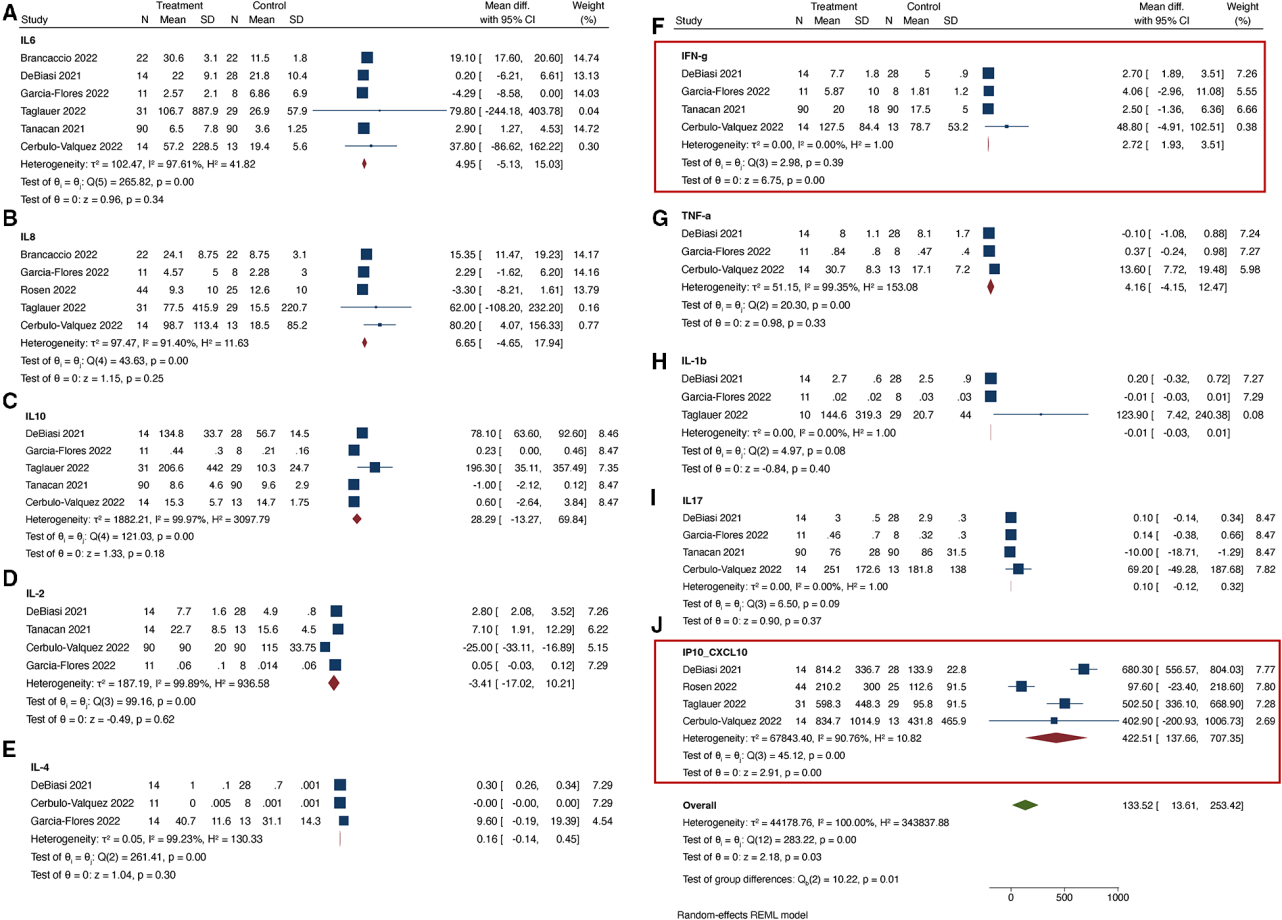
(**A–J**) Cytokines tested in maternal serum samples during acute phase of illness.

**Figure 4:**
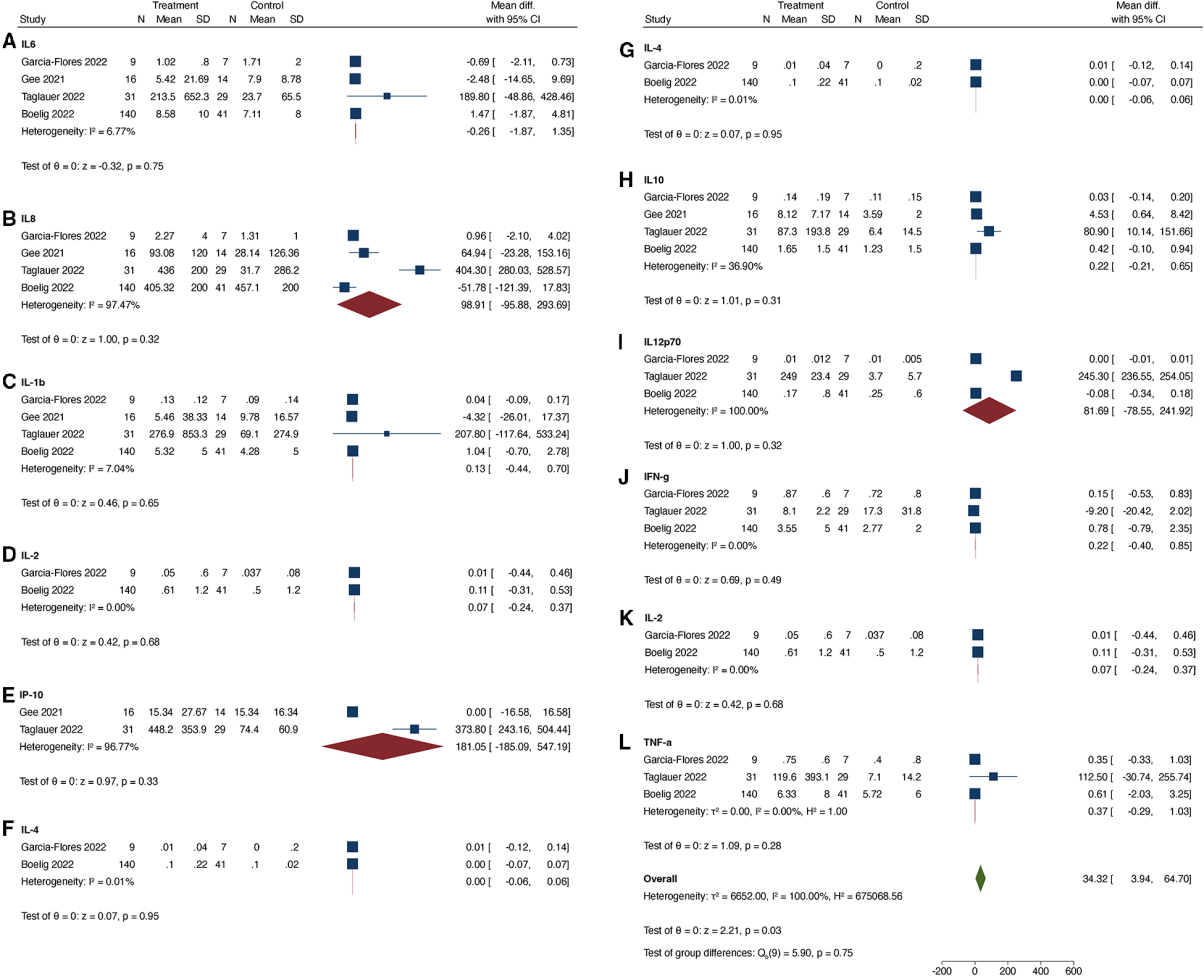
(**A–L**) Cytokines tested in cord blood samples.

When comparing COVID-19 positive pregnant women to COVID-19 negative pregnant women, we found that only the serum level of CXCL10 was significantly up-regulated in the COVID-19 positive group (Figure 2). The I^2^ statistic for majority of the cytokines was >95%, indicating significant heterogeneity among the studies. In the subset analysis, which included serum samples from COVID-19 positive women during the acute phase of illness, significant heterogeneity was also observed for most cytokines, except for IFN-γ, IL-1β, and IL-17 (Figure 3). In the subset of pregnant women with active COVID-19 during sample collection, CXCL-10 and IFN-γ were found to be significantly elevated compared to COVID-19 negative pregnant women. Finally, in the evaluation of cord blood samples, no cytokines were found to be significantly elevated in infants born to mothers with COVID-19 infection during pregnancy compared to infants born to COVID-19 negative mothers (Figure 4). There was inadequate data to conduct a subset-analysis for the cord blood samples. The 2 studies (32, 35) that reported cytokine levels in cord blood samples collected during the acute phase of maternal COVID-19 infection showed no statistically significant increase in any cytokine levels.

## Discussion

Dysregulation of the maternal immune system during pregnancy have been associated with negative pregnancy outcomes, including miscarriage, impaired fetal growth, and premature birth, which could have lasting impacts on the health of the newborns (43, 44). Amidst the COVID-19 pandemic, research has indicated elevated occurrences of preterm birth, preeclampsia, stillbirth, heightened maternal anxiety, and increased maternal mortality subsequent to SARS-CoV-2 infection during pregnancy (3–7, 45). It has been hypothesized that immune system dysregulation resulting from SARS-CoV-2 infection may mediate the observed adverse pregnancy outcomes (46). However, our meta-analysis of cytokine levels in pregnant women with and without COVID-19 infection during gestation did not demonstrate evidence of an exacerbated cytokines response in mothers with gestational COVID-19, except for elevated levels of CXCL10 and IFN-γ during the acute phase of illness. Furthermore, we found no evidence of increased cytokine levels in cord blood samples from infants exposed to COVID-19 prenatally. These findings may offer some reassurance to parents and healthcare providers caring for children born during the COVID-19 pandemic.

Systematic reviews of immune signatures secondary to COVID-19 infection in the general population have consistently reported significant elevation of cytokines like IL-6 and TNFa in severe cases (47–49). In our systematic review, we identified three studies that analyzed cytokine concentrations based on disease severity in the context of COVID-19 during pregnancy. However, none of these studies reported consistent cytokine profiles across all severity levels, preventing us from conducting a subset analysis by disease severity. Specifically, the studies by Garcia-Flores et al. (32) did not find a strong association between cytokine changes and disease severity in maternal or cord blood samples. In contrast, DB Rosen et al. (34) found that higher cytokine levels, including IL-18, IL-1Ra, IP-10, IL-2Ra were associated with more severe disease based on the NIH clinical spectrum. Tanacan et al. (36) reported statistically significant positive correlations between IFN γ and IL-6 with disease severity but observed a negative correlation for IL-2 and IL-10. These findings underscore the complexity of the relationship between cytokine profiles and disease severity in pregnant individuals with COVID-19 and emphasize the need to consider disease severity as an important variable in future studies.

Another factor impacting the measured cytokine levels is the timing of sample collection in relation to active infection. Our results did show an elevation of maternal serum levels of CXCL10 and IFN-γ during the acute phase of COVID-19 infection. CXCL10, a chemokine involved in immune cell recruitment, and IFN-γ, a pro-inflammatory cytokine, play pivotal roles in orchestrating the body's defense against viral infections. However, exaggerated immune responses may disrupt the intra-uterine environment during critical periods of development, posing potential complications for the fetus. Thus, the timing of infection during pregnancy may drive the eventual fetal outcomes. Longitudinal studies tracking these serum levels across the course of infection, as well as larger cohorts assessing the correlation between timing of COVID-19 infection during pregnancy, as well as disease severity with maternal-fetal outcomes, can provide deeper insights.

While the overall results of this meta-analysis are reassuring, they should be interpreted with caution. The included studies exhibited significant variation in terms of the timing of sample collection in relation to the onset of infection and the methods used to measure cytokine levels. Studies comparing available cytokine panels have demonstrated substantial variability in the sensitivity and specificity of these panels for detecting serum cytokine levels (50–52). Additionally, factors such as disease severity, treatment implementation, and pre-existing conditions were not adjusted for or evaluated. Moreover, it is important to note that cytokines represent only a portion of maternal immunology, and other immune markers play a crucial role in understanding the impact of SARS-CoV-2 infection on the maternal immune system. Therefore, despite our findings suggesting that SARS-CoV-2 infection during pregnancy does not result in exaggerated cytokines changes in mothers and infants, further studies are necessary to fully comprehend the risks associated with SARS-CoV-2 infection in pregnant mothers and offspring. The heterogeneity observed in this analysis underscores the need for more reliable and reproducible methods for testing serum cytokine levels, both for research and clinical purposes. Finally, it is imperative to conduct prospective investigations that involve ongoing evaluations throughout pregnancy. These studies should encompass diverse groups, considering various levels of SARS-CoV-2 disease severity and vaccination status. Such research is essential for advancing our comprehension of the connection between SARS-CoV-2 infection during pregnancy, alterations in maternal cytokines, and their enduring consequences on fetal development.

## Conclusion

This systematic review and meta-analysis of cytokine levels shows no evidence of an exacerbated or dysregulated cytokines response to SARS-CoV-2 infection in the pregnant mothers and their newborns. However, it is important to acknowledge both the strengths and limitations of this review and meta-analysis, considering the rapidly evolving nature of the literature in this field. On one hand, our study results can provide reassurance to pregnant individuals and healthcare providers caring for infants born during the COVID-19 pandemic. On the other hand, it is crucial to emphasize that our analyses should be regarded as hypothesis-generating rather than hypothesis-testing. Further research is needed to corroborate these findings and provide a more comprehensive understanding of the immune response during pregnancy in the context of SARS-CoV-2 infection. Continued monitoring and investigation of immune markers and their impact on pregnancy outcomes are warranted to inform clinical decision-making and optimize care for pregnant individuals and their infants.

## Data Availability

The original contributions presented in the study are included in the article/**Supplementary Material**, further inquiries can be directed to the corresponding author.
