# Supplementary material for: Cytokine responses to SARS-COV2 infection in mother-infant dyads: a systematic review and meta-analysis
Source: Front Pediatr. 2023 Oct 17;11:1277697. doi: 10.3389/fped.2023.1277697 (PMC10616592; doi:10.3389/fped.2023.1277697)
Supplement: Supplementary file 1 [file Table1.docx]

**Supplemental Figure 1:** Cytokines tested in maternal serum samples (additional)


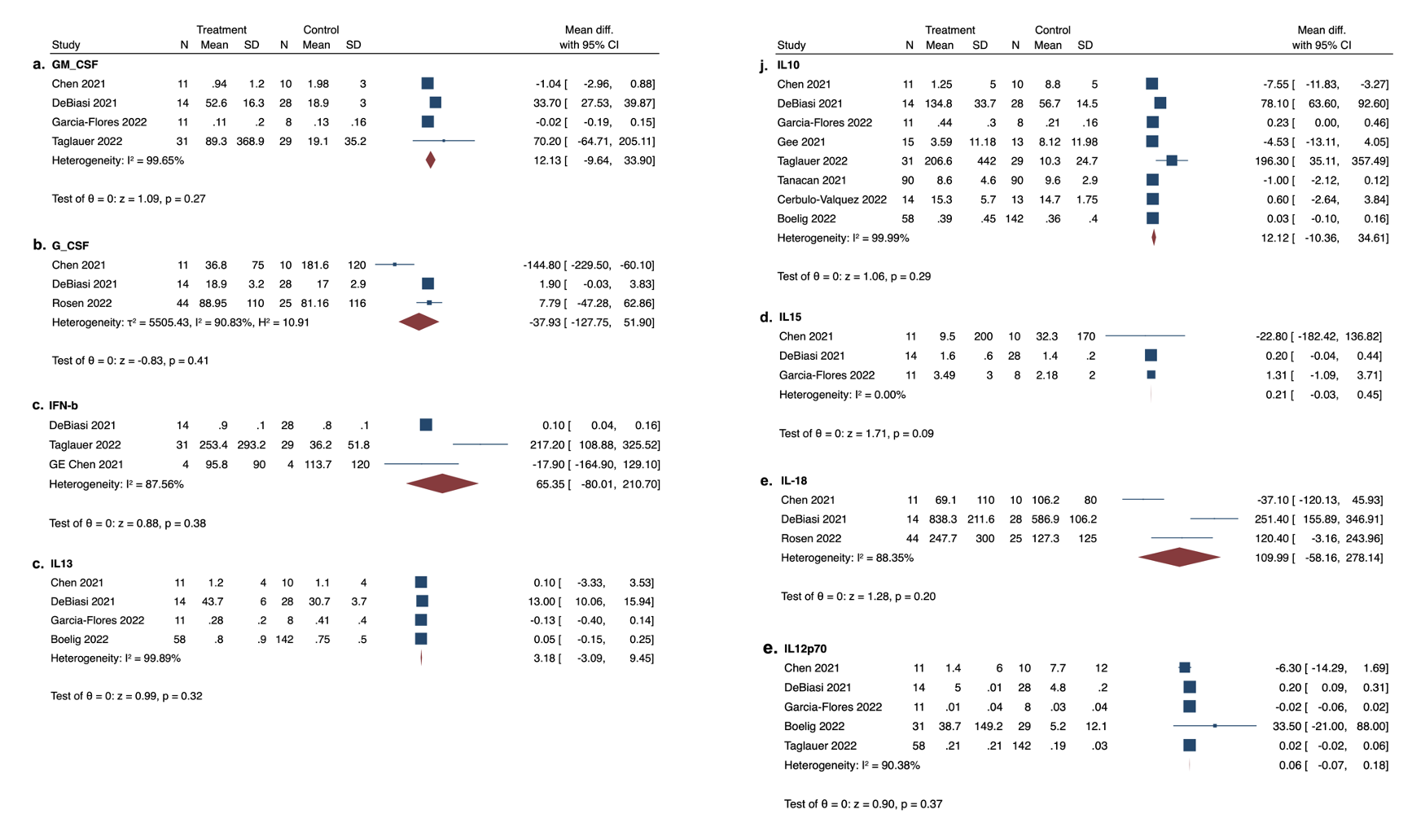


**Supplemental table 1.** Detailed information from included studies for each cytokine in maternal samples

|  |  |  |  |  |  |  |  |  |
| --- | --- | --- | --- | --- | --- | --- | --- | --- |
| **Cytokine** | **Study** | **Mean, SD/ Median, IQR (pg/ml)** | | | | | |  |
|  |  | **Study Cohort** | | | **Controls** | | | **Reported as** |
|  | Author/Year | MeanStudy | SDStudy | NumStudy | MeanControl | SDControl | NumControl |  |
| **IL-6** | Brancaccio, 2022 | 30.6 | 3.1 | 22 | 11.5 | 1.8 | 22 | Mean+SD |
|  | Boelig, 2022 | 1.32 | 1.3 | 58 | 1.1 | 0.9 | 142 | Median (IQR range) |
|  | Cerbulo-Vazquez, 2022 | 57.2 | 228.5 | 14 | 19.4 | 5.6 | 13 | Median (SD) |
|  | Chen, 2021 | 0.17 | 0.4 | 11 | 1.3 | 1.8 | 10 | Median (min-max) |
|  | De Biasi, 2021 | 22.0 | 9.1 | 14 | 21.8 | 10.4 | 28 | Mean+SEM |
|  | S Gee, 2021 | 17.33 | 45.36 | 15 | 15.16 | 29.85 | 13 | Median (IQR) |
|  | Taglauer, 2022 | 106.7 | 887.9 | 31 | 26.9 | 57.9 | 29 | Median (SD) |
|  | Tanacan, 2021 | 6.5 | 7.8 | 90 | 3.6 | 1.25 | 90 | Median (IQR) |
|  | V Garcia-Flores, 2022 | 2.57 | 2.1 | 11 | 6.86 | 6.9 | 8 | Median (IQR range) |
|  |  |  |  |  |  |  |  |  |
| **IL-8** | Brancaccio, 2022 | 24.1 | 8.75 | 22 | 8.75 | 3.1 | 22 | Mean+SD |
|  | Boelig, 2022 | 10.97 | 14 | 58 | 8.77 | 12 | 142 | Median (IQR range) |
|  | Cerbulo-Vazquez, 2022 | 98.7 | 113.4 | 14 | 18.5 | 85.2 | 13 | Median (SD) |
|  | Chen, 2021 | 12.6 | 13 | 11 | 54.8 | 59 | 10 | Median (min-max) |
|  | DB Rosen, 2022 | 9.3 | 10 | 44 | 12.6 | 10 | 25 | Median (IQR range) |
|  | S Gee, 2021 | 19.69 | 21.68 | 15 | 16.86 | 37.97 | 13 | Median (IQR) |
|  | Taglauer, 2022 | 77.5 | 415.9 | 31 | 15.5 | 220.7 | 29 | Median (SD) |
|  | V Garcia-Flores, 2022 | 4.57 | 5 | 11 | 2.28 | 3 | 8 | Median (IQR range) |
|  |  |  |  |  |  |  |  |  |
| **GM-CSF** | Chen, 2021 | 0.94 | 1.2 | 11 | 1.98 | 3 | 10 | Median (min-max) |
|  | De Biasi, 2021 | 52.6 | 16.3 | 14 | 18.9 | 3.0 | 28 | Mean+SEM |
|  | Taglauer, 2022 | 89.3 | 368.9 | 31 | 19.1 | 35.2 | 29 | Mean (SD) |
|  | V Garcia-Flores, 2022 | 0.11 | 0.2 | 11 | 0.13 | 0.16 | 8 | Median (IQR range) |
|  |  |  |  |  |  |  |  |  |
| **G-CSF** | Chen, 2021 | 36.8 | 75 | 11 | 181.6 | 120 | 10 | Median (min-max) |
|  | DB Rosen, 2022 | 88.95 | 110 | 44 | 81.16 | 116 | 25 | Median (IQR range) |
|  | De Biasi, 2021 | 18.9 | 3.2 | 14 | 17.0 | 2.9 | 28 | Mean+SEM |
|  |  |  |  |  |  |  |  |  |
| **IL-1b** | Boelig, 2022 | 1.01 | 1.2 | 58 | 0.67 | 1.27 | 142 | Median (IQR range) |
|  | Chen, 2021 | 1.58 | 5.2 | 11 | 1.4 | 1.9 | 10 | Median (min-max) |
|  | De Biasi, 2021 | 2.7 | 0.6 | 14 | 2.5 | 0.9 | 28 | Mean+SEM |
|  | S Gee, 2021 | 38.62 | 65.33 | 15 | 5.46 | 16.57 | 13 | Median (IQR) |
|  | Taglauer, 2022 | 144.6 | 319.3 | 31 | 20.7 | 44 | 29 | Mean (SD) |
|  | V Garcia-Flores, 2022 | 0.02 | 0.03 | 11 | 0.03 | 0.04 | 8 | Median (IQR range) |
|  |  |  |  |  |  |  |  |  |
| **IL1a** | Chen, 2021 | 6.1 | 13.1 | 11 | 22.5 | 15.7 | 10 | Median (min-max) |
|  | De Biasi, 2021 | 16.3 | 3.6 | 14 | 9.6 | 1.4 | 28 | Mean+SEM |
|  | V Garcia-Flores, 2022 | 1.63 | 1.1 | 11 | 0.84 | 1.1 | 8 | Median (IQR range) |
|  |  |  |  |  |  |  |  |  |
| **VEGF** | Chen, 2021 | 40.2 | 634.3 | 11 | 850 | 741.2 | 10 | Median (min-max) |
|  | De Biasi, 2021 | 104.0 | 30.9 | 14 | 72.0 | 13.1 | 28 | Mean+SEM |
|  | V Garcia-Flores, 2022 | 1.26 | 0.86 | 11 | 1.26 | 0.4 | 8 | Median (IQR range) |
|  |  |  |  |  |  |  |  |  |
| **IL12p70** | Boelig, 2022 | 0.21 | 0.2 | 58 | 0.19 | 0.4 | 142 | Median (IQR range) |
|  | Chen, 2021 | 1.4 | 8.5 | 11 | 7.7 | 7.2 | 10 | Median (min-max) |
|  | De Biasi, 2021 | 5.0 | 0.0 | 14 | 4.8 | 0.2 | 28 | Mean+SEM |
|  | Taglauer, 2022 | 38.7 | 149.2 | 31 | 5.2 | 12.1 | 29 | Mean (SD) |
|  | V Garcia-Flores, 2022 | 0.01 | 0.06 | 11 | 0.03 | 0.05 | 8 | Median (IQR range) |
|  |  |  |  |  |  |  |  |  |
| **IL-2** | Boelig, 2022 | 0.36 | 0.2 | 58 | 0.35 | 0.3 | 142 | Median (IQR range) |
|  | Cerbulo-Vazquez, 2022 | 22.7 | 8.5 | 14 | 15.6 | 4.5 | 13 | Median (SD) |
|  | Chen, 2021 | 1.8 | 9.2 | 11 | 9.8 | 5.1 | 10 | Median (min-max) |
|  | De Biasi, 2021 | 7.7 | 1.6 | 14 | 4.9 | 0.8 | 28 | Mean+SEM |
|  | Tanacan, 2021 | 90 | 20 | 90 | 115 | 33.75 | 90 | Median (IQR) |
|  | V Garcia-Flores, 2022 | 0.06 | 0.09 | 11 | 0.014 | 0.08 | 8 | Median (IQR range) |
|  |  |  |  |  |  |  |  |  |
| **IL-4** | Boelig, 2022 | 0.22 | 0.2 | 58 | 0.24 | 0.3 | 142 | Median (IQR range) |
|  | Cerbulo-Vazquez, 2022 | 40.7 | 11.6 | 14 | 31.1 | 14.3 | 13 | Median (SD) |
|  | Chen, 2021 | 3.7 | 1.1-10.7 | 11 | 1.8 | 1.1-2.3 | 10 | Median (min-max) |
|  | De Biasi, 2021 | 1.0 | 0.1 | 14 | 0.7 | 0.01 | 28 | Mean+SEM |
|  | V Garcia-Flores, 2022 | 0.0 | 0.01 | 11 | 0.0 | 0.01 | 8 | Median (IQR range) |
|  |  |  |  |  |  |  |  |  |
| **IFN-a2** | Chen, 2021 | 11.6 | 16.5 | 11 | 9.6 | 4.9 | 10 | Median (min-max) |
|  | GE Chen, 2021 | 11.7 | 1.48 | 4 | 10 | 4 | 4 | Median (IQR range) |
|  | Taglauer, 2022 | 140.7 | 442.6 | 31 | 18.1 | 34.2 | 29 | Mean (SD) |
|  |  |  |  |  |  |  |  |  |
| **IL-10** | Boelig, 2022 | 0.39 | 0.45 | 58 | 0.36 | 0.4 | 142 | Median (IQR range) |
|  | Cerbulo-Vazquez, 2022 | 15.3 | 5.7 | 14 | 14.7 | 1.75 | 13 | Median (SD) |
|  | Chen, 2021 | 1.25 | 5 | 11 | 8.8 | 5 | 10 | Median (min-max) |
|  | De Biasi, 2021 | 134.8 | 33.7 | 14 | 56.7 | 14.5 | 28 | Mean+SEM |
|  | S Gee, 2021 | 3.59 | 11.18 | 15 | 8.12 | 11.98 | 13 | Median (IQR) |
|  | Taglauer, 2022 | 206.6 | 442 | 31 | 10.3 | 24.7 | 29 | Mean (SD) |
|  | Tanacan, 2021 | 8.6 | 4.6 | 90 | 9.6 | 2.9 | 90 | Median (IQR) |
|  | V Garcia-Flores, 2022 | 0.44 | 0.3 | 11 | 0.21 | 0.16 | 8 | Median (IQR range) |
|  |  |  |  |  |  |  |  |  |
| **IL-13** | Boelig, 2022 | 0.8 | 0.9 | 58 | 0.75 | 0.5 | 142 | Median (IQR range) |
|  | Chen, 2021 | 1.2 | 4 | 11 | 1.1 | 4 | 10 | Median (min-max) |
|  | De Biasi, 2021 | 43.7 | 6.0 | 14 | 30.7 | 3.7 | 28 | Mean+SEM |
|  | V Garcia-Flores, 2022 | 0.28 | 0.2 | 11 | 0.41 | 0.4 | 8 | Median (IQR range) |
|  |  |  |  |  |  |  |  |  |
| **IL-17** | Cerbulo-Vazquez, 2022 | 251 | 172.6 | 14 | 181.8 | 138 | 13 | Median (SD) |
|  | Chen, 2021 | 5.3 | 12 | 11 | 9.3 | 8 | 10 | Median (min-max) |
|  | De Biasi, 2021 | 3.0 | 0.5 | 14 | 2.9 | 0.3 | 28 | Mean+SEM |
|  | Tanacan, 2021 | 76 | 28 | 90 | 86 | 31.5 | 90 | Median (IQR) |
|  | V Garcia-Flores, 2022 | 0.46 | 0.7 | 11 | 0.32 | 0.3 | 8 | Median (IQR range) |
|  |  |  |  |  |  |  |  |  |
| **IP-10/ CXCL-10** | Cerbulo-Vazquez, 2022 | 834.7 | 1014.9 | 14 | 431.8 | 465.9 | 13 | Median (SD) |
|  | Chen, 2021 | 506.3 | 600 | 11 | 417.7 | 556.9 | 10 | Median (min-max) |
|  | DB Rosen, 2022 | 210.2 | 300 | 44 | 112.6 | 91.5 | 25 | Median (IQR range) |
|  | De Biasi, 2021 | 814.2 | 336.7 | 14 | 133.9 | 22.8 | 28 | Mean+SEM |
|  | S Gee, 2021 | 37.96 | 197.07 | 15 | 19.11 | 10.42 | 13 | Median (IQR) |
|  | Taglauer, 2022 | 598.3 | 448.3 | 31 | 95.8 | 91.5 | 29 | Median (SD) |
|  |  |  |  |  |  |  |  |  |
| **IL-15** | Chen, 2021 | 9.5 | 200 | 11 | 32.3 | 170 | 10 | Median (min-max) |
|  | De Biasi, 2021 | 1.6 | 0.6 | 14 | 1.4 | 0.2 | 28 | Mean+SEM |
|  | V Garcia-Flores, 2022 | 3.49 | 3 | 11 | 2.18 | 240 | 8 | Median (IQR range) |
|  |  |  |  |  |  |  |  |  |
| **TNF-a** | Boelig, 2022 | 2.54 | 2.5 | 58 | 2.36 | 2.25 | 142 | Median (IQR range) |
|  | Cerbulo-Vazquez, 2022 | 30.7 | 8.3 | 14 | 17.1 | 7.2 | 13 | Median (SD) |
|  | Chen, 2021 | 69.2 | 17.3-157.7 | 11 | 59.6 | 50 | 10 | Median (min-max) |
|  | De Biasi, 2021 | 8.0 | 1.1 | 14 | 8.1 | 1.7 | 28 | Mean+SEM |
|  | Febryanna | 3.42 | 7.24 | 25 | 2.70 | 3.06 | 25 | Median (IQR) |
|  | V Garcia-Flores, 2022 | 0.84 | 0.8 | 11 | 0.47 | 0.4 | 8 | Median (IQR range) |
|  |  |  |  |  |  |  |  |  |
| **IFN-g** | Boelig, 2022 | 2.23 | 2 | 58 | 2.2 | 2 | 142 | Median (IQR range) |
|  | Cerbulo-Vazquez, 2022 | 127.5 | 84.4 | 14 | 78.7 | 53.2 | 13 | Median (SD) |
|  | Chen, 2021 | 43.4 | 30 | 11 | 36.8 | 20.75-59.4 | 10 | Median (min-max) |
|  | De Biasi, 2021 | 7.7 | 1.8 | 14 | 5.0 | 0.9 | 28 | Mean+SEM |
|  | GE Chen, 2021 | 26.7 | 31 | 4 | 40 | 35 | 4 | Median (IQR range) |
|  | Taglauer, 2022 | 144.6 | 319.3 | 31 | 20.7 | 44 | 29 | Mean (SD) |
|  | Tanacan, 2021 | 20 | 18 | 90 | 17.5 | 5 | 90 | Median (IQR) |
|  | V Garcia-Flores, 2022 | 5.87 | 10 | 11 | 1.81 | 1.2 | 8 | Median (IQR range) |
|  |  |  |  |  |  |  |  |  |
| **IL-18** | Chen, 2021 | 69.1 | 110 | 11 | 106.2 | 80 | 10 | Median (min-max) |
|  | DB Rosen, 2022 | 247.7 | 300 | 44 | 127.3 | 125 | 25 | Median (IQR) |
|  | De Biasi, 2021 | 838.3 | 211.6 | 14 | 586.9 | 106.2 | 28 | Mean+SEM |
|  |  |  |  |  |  |  |  |  |
| **IFN-b** | De Biasi, 2021 | 0.9 | 0.0 | 14 | 0.8 | 0.0 | 28 | Mean+SEM |
|  | GE Chen, 2021 | 95.8 | 90 | 4 | 113.7 | 120 | 4 | Median (IQR range) |
|  | Taglauer, 2022 | 253.4 | 293.2 | 31 | 36.2 | 51.8 | 29 | Mean (SD) |
|  |  |  |  |  |  |  |  |  |

**Supplemental Table 2.** Detailed information from included studies for each cytokine in cord blood samples.

|  |  |  | | | | | |  |
| --- | --- | --- | --- | --- | --- | --- | --- | --- |
|  |  | **Mean, SD/ Median, IQR (pg/ml)** | | | | | |  |
| **Cytokine** | **Study** | **Study cohort** | | | **Controls** | | | **Reported as** |
|  | Author/Year | MeanInt | SDInt | NumInt | MeanControl | SDControl | NumControl |  |
| **IL-6** | Boelig, 2022 | 8.58 | 10 | 41 | 7.11 | 8 | 140 | Median (IQR range) |
|  | S Gee, 2021 | 5.42 | 21.69 | 16 | 7.9 | 8.78 | 14 | Median (IQR) |
|  | Taglauer, 2022 | 213.5 | 652.3 | 31 | 23.7 | 65.5 | 29 | Median (SD) |
|  | V Garcia-Flores, 2022 | 1.02 | 0.8 | 9 | 1.71 | 2 | 7 | Median (IQR range) |
|  |  |  |  |  |  |  |  |  |
| **IFN-g** | Boelig, 2022 | 3.55 | 5 | 140 | 2.77 | 2 | 41 | Median (IQR range) |
|  | Taglauer, 2022 | 8.1 | 2.2 | 31 | 17.3 | 31.8 | 29 | Mean (SD) |
|  | V Garcia-Flores, 2022 | 0.87 | 0.6 | 9 | 0.72 | 0.8 | 7 | Median (IQR range) |
|  |  |  |  |  |  |  |  |  |
| **IL-1b** | Boelig, 2022 | 5.32 | 5 | 140 | 4.28 | 5 | 41 | Median (IQR range) |
|  | S Gee, 2021 | 5.46 | 38.33 | 16 | 9.78 | 16.57 | 14 | Median (IQR) |
|  | Taglauer, 2022 | 276.9 | 853.3 | 31 | 69.1 | 274.9 | 29 | Mean (SD) |
|  | V Garcia-Flores, 2022 | 0.13 | 0.12 | 9 | 0.09 | 0.14 | 7 | Median (IQR range) |
|  |  |  |  |  |  |  |  |  |
| **IL-8** | Boelig, 2022 | 405.32 | 405.32 | 140 | 457.1 | 200 | 41 | Median (IQR range) |
|  | S Gee, 2021 | 93.08 | 262.63 | 16 | 28.14 | 126.36 | 14 | Median (IQR) |
|  | Taglauer, 2022 | 636.2 | 5631.6 | 31 | 31.7 | 286.2 | 29 | Median (SD) |
|  | V Garcia-Flores, 2022 | 2.27 | 4 | 9 | 1.31 | 1 | 7 | Median (IQR range) |
|  |  |  |  |  |  |  |  |  |
| **IL-10** | Boelig, 2022 | 1.65 | 1.5 | 140 | 1.23 | 1.5 | 41 | Median (IQR range) |
|  | S Gee, 2021 | 8.12 | 7.17 | 16 | 3.59 | 0 | 14 | Median (IQR) |
|  | Taglauer, 2022 | 87.3 | 193.8 | 31 | 6.4 | 14.5 | 29 | Mean (SD) |
|  | V Garcia-Flores, 2022 | 0.14 | 0.19 | 9 | 0.11 | 0.15 | 7 | Median (IQR range) |
|  |  |  |  |  |  |  |  |  |
| **IL12p70** | Boelig, 2022 | 0.17 | 0.8 | 140 | 0.25 | 0.6 | 41 | Median (IQR range) |
|  | Taglauer, 2022 | 249 | 23.4 | 31 | 3.7 | 5.7 | 29 | Mean (SD) |
|  | V Garcia-Flores, 2022 | 0.01 | 0.012 | 9 | 0.01 | 0.05 | 7 | Median (IQR range) |
|  |  |  |  |  |  |  |  |  |
| **TNF-a** | Boelig, 2022 | 6.33 | 8 | 140 | 5.72 | 6 | 41 | Median (IQR range) |
|  | Taglauer, 2022 | 119.6 | 393.1 | 31 | 7.1 | 14.2 | 29 | Mean (SD) |
|  | V Garcia-Flores, 2022 | 0.75 | 0.6 | 9 | 0.4 | 0.8 | 7 | Median (IQR range) |
|  |  |  |  |  |  |  |  |  |
| **IP-10** | S Gee, 2021 | 15.34 | 27.67 | 16 | 15.34 | 16.34 | 14 | Median (IQR) |
|  | Taglauer, 2022 | 448.2 | 353.9 | 31 | 74.4 | 60.9 | 29 | Mean (SD) |
|  |  |  |  |  |  |  |  |  |
| **IL-2** | Boelig, 2022 | 0.61 | 1.2 | 140 | 0.5 | 1.2 | 41 | Median (IQR range) |
|  | V Garcia-Flores, 2022 | 0.05 | 0.6 | 9 | 0.037 | 0.08 | 7 | Median (IQR range) |
|  |  |  |  |  |  |  |  |  |
| **IL-4** | Boelig, 2022 | 0.1 | 0.22 | 140 | 0.1 | 0.02 | 41 | Median (IQR range) |
|  | V Garcia-Flores, 2022 | 0.01 | 0.04 | 9 | 0 | 0.2 | 7 | Median (IQR range) |

**Supplemental table 3.** Detailed information from studies included in subset analysis for each cytokine in maternal samples.

|  |  |  |  |  |  |  |  | |  |
| --- | --- | --- | --- | --- | --- | --- | --- | --- | --- |
| **Cytokine** | **Author** | **Mean, SD/ Median, IQR (pg/ml)** | | | | | |  |  |
|  |  | **Study Cohort** | | | **Controls** | | | | **Reported as** |
|  | Author | MeanStudy | SDStudy | NumStudy | MeanControl | SDControl | NumControl | |  |
| **IL-6** | Brancaccio, 2022 | 30.6 | 3.1 | 22 | 11.5 | 1.8 | 22 | | Mean+SD |
|  | Cerbulo-Vazquez, 2022 | 57.2 | 228.5 | 14 | 19.4 | 5.6 | 13 | | Median (SD) |
|  | De Biasi, 2021 | 22 | 9.1 | 14 | 21.8 | 10.4 | 28 | | Mean+SEM |
|  | Taglauer, 2022 | 77.12 | 462.2 | 10 | 26.9 | 57.9 | 28 | | Median (SD) |
|  | Tanacan, 2021 | 6.5 | 7.8 | 90 | 3.6 | 1.25 | 90 | | Median (IQR) |
|  | V Garcia-Flores, 2022 | 2.57 | 2.1 | 11 | 6.86 | 6.9 | 8 | | Median (IQR range) |
|  |  |  |  |  |  |  |  | |  |
| **IL-8** | Brancaccio, 2022 | 24.1 | 8.75 | 22 | 8.75 | 3.1 | 22 | | Mean+SD |
|  | Cerbulo-Vazquez, 2022 | 98.7 | 113.4 | 14 | 18.5 | 85.2 | 13 | | Median (SD) |
|  | DB Rosen, 2022 | 17.24 | 34.6 | 14 | 22.98 | 25.7 | 25 | | Median (IQR range) |
|  | Taglauer, 2022 | 71 | 232.25 | 10 | 47.3 | 392.81 | 29 | | Median (SD) |
|  | V Garcia-Flores, 2022 | 4.57 | 3.4 | 11 | 2.28 | 4.3 | 8 | | Median (IQR range) |
|  |  |  |  |  |  |  |  | |  |
| **IL-1b** | De Biasi, 2021 | 2.7 | 0.6 | 14 | 2.5 | 0.9 | 28 | | Mean+SEM |
|  | Taglauer, 2022 | 144.6 | 319.3 | 10 | 20.7 | 44 | 29 | | Mean (SD) |
|  | V Garcia-Flores, 2022 | 0.02 | 0.02 | 11 | 0.03 | 0.03 | 8 | | Median (IQR range) |
|  |  |  |  |  |  |  |  | |  |
| **IL-2** | Cerbulo-Vazquez, 2022 | 22.7 | 8.5 | 14 | 15.6 | 4.5 | 13 | | Median (SD) |
|  | De Biasi, 2021 | 7.7 | 1.6 | 14 | 4.9 | 0.8 | 28 | | Mean+SEM |
|  | Tanacan, 2021 | 90 | 20 | 90 | 115 | 33.75 | 90 | | Median (IQR) |
|  | V Garcia-Flores, 2022 | 0.06 | 0.1 | 11 | 0.014 | 0.06 | 8 | | Median (IQR range) |
|  |  |  |  |  |  |  |  | |  |
| **IL-4** | Cerbulo-Vazquez, 2022 | 40.7 | 11.6 | 14 | 31.1 | 14.3 | 13 | | Median (SD) |
|  | De Biasi, 2021 | 1.0 | 0.1 | 14 | 0.7 | 0.0 | 28 | | Mean+SEM |
|  | V Garcia-Flores, 2022 | 0.01 | 0.05 | 11 | 0.01 | 0.01 | 8 | | Median (IQR range) |
|  |  |  |  |  |  |  |  | |  |
| **IL-10** | De Biasi, 2021 | 134.8 | 33.7 | 14 | 56.7 | 14.5 | 28 | | Mean+SEM |
|  | Cerbulo-Vazquez, 2022 | 15.3 | 5.7 | 14 | 14.7 | 1.75 | 13 | | Median (SD) |
|  | Taglauer, 2022 | 73.6 | 214.9 | 10 | 10.3 | 24.7 | 29 | | Mean (SD) |
|  | Tanacan, 2021 | 8.6 | 4.6 | 90 | 9.6 | 2.9 | 90 | | Median (IQR) |
|  | V Garcia-Flores, 2022 | 0.44 | 0.3 | 11 | 0.21 | 0.16 | 8 | | Median (IQR range) |
|  |  |  |  |  |  |  |  | |  |
| **IL-17** | Cerbulo-Vazquez, 2022 | 251 | 172.6 | 14 | 181.8 | 138 | 13 | | Median (SD) |
|  | De Biasi, 2021 | 3.0 | 0.5 | 14 | 2.9 | 0.3 | 28 | | Mean+SEM |
|  | Tanacan, 2021 | 76 | 28 | 90 | 86 | 31.5 | 90 | | Median (IQR) |
|  | V Garcia-Flores, 2022 | 0.46 | 0.7 | 11 | 0.32 | 0.3 | 8 | | Median (IQR range) |
|  |  |  |  |  |  |  |  | |  |
| **IP-10/ CXCL-10** | Cerbulo-Vazquez, 2022 | 834.7 | 1014.9 | 14 | 431.8 | 465.9 | 13 | | Median (SD) |
|  | DB Rosen, 2022 | 49.99 | 40.8 | 14 | 12.5 | 3.5 | 25 | | Median (IQR range) |
|  | De Biasi, 2021 | 814.2 | 336.7 | 14 | 133.9 | 22.8 | 28 | | Mean+SEM |
|  | Taglauer, 2022 | 598.3 | 448.3 | 10 | 95.8 | 91.5 | 29 | | Median (SD) |
|  |  |  |  |  |  |  |  | |  |
| **TNF-a** | Cerbulo-Vazquez, 2022 | 30.7 | 8.3 | 14 | 17.1 | 7.2 | 13 | | Median (SD) |
|  | De Biasi, 2021 | 8.0 | 1.1 | 14 | 8.1 | 1.7 | 28 | | Mean+SEM |
|  | V Garcia-Flores, 2022 | 0.84 | 0.8 | 11 | 0.47 | 0.4 | 8 | | Median (IQR range) |
|  |  |  |  |  |  |  |  | |  |
| **IFN-g** | Cerbulo-Vazquez, 2022 | 127.5 | 84.4 | 14 | 78.7 | 53.2 | 13 | | Median (SD) |
|  | De Biasi, 2021 | 7.7 | 1.8 | 14 | 5.0 | 0.9 | 28 | | Mean+SEM |
|  | Tanacan, 2021 | 20 | 18 | 90 | 17.5 | 5 | 90 | | Median (IQR) |
|  | V Garcia-Flores, 2022 | 5.87 | 10 | 11 | 1.81 | 1.2 | 8 | | Median (IQR range) |
|  |  |  |  |  |  |  |  | |  |

**Supplemental document 1. Search methods details (search strings)**

Covid & pregnancy

Date searched: 8/29/22

Limits: In Embase limit to articles/articles in press

PubMed:

("COVID-19"[Mesh] OR “Covid-19”[tiab] OR "SARS-CoV-2"[Mesh] OR SARS-CoV-2[tiab] OR "Coronavirus"[Mesh] OR coronavirus[tiab])

AND (pregnancy[tiab] OR “Pregnancy”[Mesh] OR “pregnancy complications”[tiab] OR “Pregnancy Complications”[Mesh] OR maternal[tiab] OR “maternal health”[tiab])

AND ("immunology" [Subheading] OR immunolog*[tiab] OR cytokines[tiab] OR "Cytokines"[Mesh] OR inflammation[tiab] OR inflammatory[tiab] OR “inflammatory profile”[tiab] OR "Inflammation Mediators"[Mesh] OR “inflammation mediators”[tiab])

References = 933

Web of Science:

(“Covid-19” OR SARS-CoV-2 OR coronavirus) AND (pregnancy OR “pregnancy complications” OR maternal OR “maternal health”) AND (immunolog* OR cytokines OR inflammation OR inflammatory OR “inflammatory profile” OR "Inflammation Mediators")

References = 666

Embase:

('coronavirus disease 2019'/exp OR 'coronavirus disease 2019' OR 'coronavirinae'/exp OR 'coronavirinae') AND ('pregnancy'/exp OR 'pregnancy' OR 'pregnancy complication'/exp OR 'pregnancy complication' OR 'maternal'/exp OR 'maternal' OR 'maternal care'/exp OR 'maternal care') AND ('immunology'/exp OR 'immunology' OR 'cytokine'/exp OR 'cytokine' OR 'inflammation'/exp OR 'inflammation' OR 'inflammatory profile' OR 'inflammatory protein'/exp OR 'inflammatory protein')

References = 1,420

Coronavirus Research Database:

(“Covid-19” OR SARS-CoV-2 OR coronavirus) AND (pregnancy OR “pregnancy complications” OR maternal OR “maternal health”) AND (immunolog* OR cytokines OR inflammation OR inflammatory OR “inflammatory profile” OR "Inflammation Mediators")

References = 216

Total references from all databases = 3,235

Total references after duplicates removed = 2,617*

Total references after manual duplicates removed = 2074

Excluded after title and abstract review= 1809

Full text review= 265

Excluded after full text review= 248

Papers included= 17
